# Simple spatio-temporal transformation with sub-threshold integration in the saccadic system

**DOI:** 10.1186/1471-2202-12-S1-P133

**Published:** 2011-07-18

**Authors:** Emmanuel Daucé, Anthony Mouraud, Alain Guillaume

**Affiliations:** 1 Institut des Sciences du Mouvement, CNRS UMR 6233, Marseille, France; 2 CEA, Saclay, France; 3 Laboratoire de Neurobiologie de la Cognition, CNRS UMR 6155, Marseille, France

## 

Saccades are the fast eye movements dedicated to sight orientation toward targets. Robinson [1] suggested a model based on feedback control according to an internally estimated motor error relying on a "neuronal integrator". Although supported by numerous behavioral studies, this principle is still in search of biological confirmation. Indeed, neurophysiological data has not yet uncovered all the details of the complex circuitry supporting saccade control. The displacement command is monitored by a brief pulse of activity emitted by saccadic burst neurons of the brain stem resulting in a stereotypic duration/velocity relationship (the main sequence). Burst neurons are under the control of the deep layers of the superior colliculus (SC) which encode saccade command as a focal burst of activity on a bidimensional retinotopic map. The transformation from bidimensional activity toward a precise burst is called the spatio-temporal transformation [2]. The many functional models that have been proposed [3] reproduce most of the robustness to perturbation observed, but lack in neuronal implementation details. Most of all, the "neuronal integrator" appears delicate to simulate with realistic neurons.

Our proposition is that this integration component could simply rely on neuronal sub-threshold membrane integration with appropriate membrane time constant. The model we present is built with realistic spiking neurons (excitatory and inhibitory LIF neurons with reverse potentials - Damned simulator [4]). The general scheme is inspired from simple principles exposed in [5] suggesting that duration and velocity may be coded by two independent tracks from the colliculus (Dual path principle) [6].

The model comprise a complete SC topographic map and the path from the map to premotor burst neurons, including central mesencephalic reticular formation (cMRF) and omni-pause neurons (OPN). With appropriate projections, saccadic pre-motor activity is produced at the output of the system. Most of state-of-the-art properties are obtained :spatio-temporal transformation, component stretching, staircase saccades, etc. Our model also suggests distinct roles taken by ipsilateral and contralateral cMRF: contralateral cMRF could play the role of a "gate" silencing the OPN and allowing the burst neurons to start spiking, while ipsilateral cMRF would integrate the signal from SC and emit a brief burst of spikes at the end of saccade, as observed in [7].
